# The spatiotemporal distribution of historical malaria cases in Sweden: a climatic perspective

**DOI:** 10.1186/s12936-021-03744-9

**Published:** 2021-05-01

**Authors:** Tzu Tung Chen, Fredrik Charpentier Ljungqvist, Helene Castenbrandt, Franziska Hildebrandt, Mathias Mølbak Ingholt, Jenny C. Hesson, Johan Ankarklev, Kristina Seftigen, Hans W. Linderholm

**Affiliations:** 1 Regional Climate Group, Department of Earth Sciences, University of Gothenburg, 405 30 Gothenburg, Sweden; 2 Department of History, Stockholm University, 106 91 Stockholm, Sweden; 3 Bolin Centre for Climate Research, Stockholm University, 106 91 Stockholm, Sweden; 4 Swedish Collegium for Advanced Study, Linneanum, Thunbergsvägen 2, 752 38 Uppsala, Sweden; 5 Department of Economic History, Lund University, Lund, Sweden; 6 Department of Molecular Biosciences, The Wenner-Gren Institute, Stockholm University, Stockholm, Sweden; 7 PandemiX Center, Department of Science and Environment, Roskilde University, Roskilde, Denmark; 8 Zoonosis Science Center, Department of Medical Biochemistry and Microbiology, Uppsala University, Uppsala, Sweden; 9 Dendro Sciences Group, Swiss Federal Research Institute WSL, Birmensdorf, Switzerland

**Keywords:** Malaria, *Plasmodium vivax*, Epidemic, History, Infectious disease, GIS, Summer temperature, Summer precipitation, Sweden

## Abstract

**Background:**

Understanding of the impacts of climatic variability on human health remains poor despite a possibly increasing burden of vector-borne diseases under global warming. Numerous socioeconomic variables make such studies challenging during the modern period while studies of climate–disease relationships in historical times are constrained by a lack of long datasets. Previous studies have identified the occurrence of malaria vectors, and their dependence on climate variables, during historical times in northern Europe. Yet, malaria in Sweden in relation to climate variables is understudied and relationships have never been rigorously statistically established. This study seeks to examine the relationship between malaria and climate fluctuations, and to characterise the spatio-temporal variations at parish level during severe malaria years in Sweden 1749–1859.

**Methods:**

Symptom-based annual malaria case/death data were obtained from nationwide parish records and military hospital records in Stockholm. Pearson (r_p_) and Spearman’s rank (r_s_) correlation analyses were conducted to evaluate inter-annual relationship between malaria data and long meteorological series. The climate response to larger malaria events was further explored by Superposed Epoch Analysis, and through Geographic Information Systems analysis to map spatial variations of malaria deaths.

**Results:**

The number of malaria deaths showed the most significant positive relationship with warm-season temperature of the preceding year. The strongest correlation was found between malaria deaths and the mean temperature of the preceding June–August (r_s_ = 0.57, p < 0.01) during the 1756–1820 period. Only non-linear patterns can be found in response to precipitation variations. Most malaria hot-spots, during severe malaria years, concentrated in areas around big inland lakes and southern-most Sweden.

**Conclusions:**

Unusually warm and/or dry summers appear to have contributed to malaria epidemics due to both indoor winter transmission and the evidenced long incubation and relapse time of *P. vivax*, but the results also highlight the difficulties in modelling climate–malaria associations. The inter-annual spatial variation of malaria hot-spots further shows that malaria outbreaks were more pronounced in the southern-most region of Sweden in the first half of the nineteenth century compared to the second half of the eighteenth century.

## Introduction

Changes in climate, as well as in the frequency and duration of extreme weather events directly affect human health, e.g. by increased mortality during heatwaves [1]. Even comparably small changes in temperature and precipitation can result in measurable impacts on diseases [2]. Although many health risks are associated with secondary climatic influences, such as shifting patterns of disease vectors or affects on food production, water supplies, social disruption, and migration [3–5], climate is ranked as one of the most important drivers of infectious disease [6]. Hence, studies of human health vulnerability in relation to changing climate or extreme weather events need to include both direct and indirect impacts of climate change [7].

The distribution and abundance of hosts and vectors, and the transmitted pathogens of vector-borne diseases, are strongly influenced by changes in temperature and precipitation [8, 9]. Evidences of outbreaks driven by temperature anomalies in Europe [10], and projected spread of vectors with the presence of warming [11], indicated that the expected increase in global mean temperature, and associated changes in regional climate patterns, will very likely have effects on human health and on the prevalence and distribution of various vector-borne diseases [12].

One of the major human-infecting vector-borne diseases today, malaria, is described to have had a huge impact on the evolutionary selection of the human genome [13, 14], and it has historically caused the largest amount of human mortality among all infectious diseases [15]. Malaria is caused by the replication of parasites of the genus *Plasmodium* in the human blood and transmitted between humans by mosquitoes of the genus *Anopheles*. Today malaria is almost exclusively found in tropical regions, with an estimated present-day prevalence of more than 200 million cases per year [16]. However, up until the early twentieth century, malaria also plagued the population in regions with a temperate climate. It was even endemic up to sub-Arctic regions in e.g. Finland and European Russia.

Malaria was historically established throughout much of the European continent at the latest during Roman times [17], and spread to new regions with trade and shipping [18]. Since at least medieval times, malaria has remained endemic in Sweden, with evidence pointing to the existence of a former Scandinavian strain of the malaria parasite, *Plasmodium vivax* [19]. This *P. vivax* strain phenotype was described to have a prolonged incubation time of 8–10 months until primary infection, and long latency phases of up to 9 years until relapse were commonly reported [20, 21]. This Scandinavian *P. vivax* strain, which is extinct today, was presumably closely related to the *P. vivax hibernans* strain, isolated in Russia in 1949 [20, 22].

In addition, *Plasmodium malariae* was reported in parts of Europe [23] and causes quartan malaria [24], which has been reported among other forms of malaria in Sweden [25]. This either indicates that the Scandinavian *Plasmodium* strain shared characteristics of different currently described *Plasmodium* species, or the presence of different *Plasmodium* species in Sweden. In 1982 the dormant hypnozoite stage of *P. vivax*, permitting the relapse of malaria, was first identified [26]. This unique biology of *P. vivax* has its epidemiology distinct from *P. falciparum* in Africa which has short incubation time and relapse interval [27], and the ability to develop in colder temperatures further adds to its predominance in wider geographic range. However, molecular cues inducing relapse of *P. vivax* from hypnozoites to the symptomatic erythrocytic stage remain largely unknown. Several hypotheses have been proposed to explain *P. vivax*’s survival during hibernation and the transmission of the parasite after winter in regions with highly seasonal climate such as northern Europe.

Importantly, recent findings suggest that *Plasmodium* parasites are able to adjust infection and relapse behavior to the presence of endemic mosquitoes which is indirectly dictated by climatic conditions [20, 21, 28]. A study about malaria in Finland during the eighteenth and nineteenth centuries proposed that *P. vivax* potentially detects the presence of *Anopheles* vectors and thereby adjusts transmission timing. This would allow the parasite to stay in the dormant hypnozoite state until vector season, circumventing transmission limitations by unstable weather conditions [20]. However, empirical experiments to evaluate the validity of this hypothesis remains to be performed. Additionally, the survival and spread of the *Plasmodium* parasite were possibly facilitated by co-habitation of infected, hibernating mosquitoes in strongly climate-controlled environments together with humans during the winter season [29]. Thus, the mosquito vector plays a crucial role in the understanding of historical seasonality, infection and relapse of *P. vivax* malaria in northern Europe.

In Sweden, the genus *Anopheles* is represented by eight species [30, 31]. The species that historically have been involved in malaria transmission belong to the *An. maculipennis* complex, which consists of closely related species that are difficult to identify based on morphological characters [32]. This includes the species *Anopheles* (*Anopheles*) *messeae* Falleroni 1926, which was considered to have been the main vector of malaria in Sweden as well as other countries with similar climatic conditions such as Finland, parts of Russia and other regions of the former Soviet Union [20, 30, 32, 33]. It also includes *Anopheles* (*Anopheles*) *atroparvus* van Thiel 1927, which is the proposed vector of malaria in the coastal regions of southern Sweden due to its preference to brackish larval habitat [32].

Both of these *Anopheles* species spend the winter season as inseminated females, e.g. in stables. At emergence in spring (i.e. March–April in southern Sweden) they will blood feed and thereafter lay their eggs in stagnant water bodies, such as flood-plains, edges of rivers and lakes, ditches, and ponds [34]. The first adults of the summer generation usually emerge in June and they prefer to rest and blood-feed indoors on large domestic animals, e.g. livestock, but will occasionally also feed on humans. Some females of this generation may, depending on temperature and humidity, survive long enough for the malaria parasite to complete its development and thereby function as malaria vectors in late summer [34]. Eggs produced by these females will hatch into the coming overwintering generation. In winter, *Anopheles atroparvus* is known for taking irregular blood-meals while the behaviour of *Anopheles messeae* has been reported to vary; at colder sites it remains inactive while it may take irregular blood-meals when overwintering at warmer sites [33, 34]. Thus, this blood-feeding behaviour may enable transmission of the malaria parasite within households during winter times. It has been proposed that *Plasmodium* sporozoites can alter the behaviour of infected mosquitoes, leading to increased blood-feeding behavior which would optimize transmission during the winter months [33].

Change and variability of climate affects human health and the spread of diseases, in various ways, on intra-annual to multi-decadal, and even centennial, time-scales [35]. Except for weather-related events at inter-annual time-scales, decades with warm summers appear to coincide with peaks of malaria incidence across northern Europe [36, 37]. It was concluded that summer temperature was determinant for mosquito population, consequently reflecting transmission frequency. Yet, from a long-term perspective, malaria is capable of transmitting indoors throughout the year despite the presence of cold weather and strong climatic fluctuations.

Malaria in Sweden has been eliminated. Today malaria only occurs as imported cases in Sweden [38], a situation similar to elsewhere in Europe [39–42]. The decrease in malaria transmission has been mainly attributed to improved socioeconomic conditions, which included better healthcare (the increase in treatment of quinine [43]) and better hygiene conditions (draining of wetlands [34]). Although it has been speculated that the comparably low summer temperatures during the eighteenth and nineteenth centuries perhaps also played a role in the long-term decline in malaria transmission [43, 44], this explanation appears highly questionable since palaeoclimate records show even colder conditions during the seventeenth century [5, 45, 46]. Notably, long-term warming at higher latitudes have not shown decisive influences on malaria dynamics in the long run [29, 36] as has been predicted by some malaria models [47].

Among the Nordic countries, the disease has been best studied in Finland, where a strong relationship between malaria outbreaks and preceding summer temperature was established for the nineteenth century [29]. However, except for Finland, data in the other Nordic countries are not fully explored in the distributions of malaria in modern studies, and the status of the malaria history of Sweden is obsolete. Thus, the aim of this study is to examine the effects of short-term climate fluctuations on malaria based on historical data from Sweden, and with the application of GIS tools to trace the spatio-temporal variations of malaria hot-spot, which can provide useful information to investigations of controlling socioeconomic factors. In this study, the link between temperature and the inter-annual to decadal dynamics of malaria is statistically established, and reveal the regional variation of more severe malaria epidemics at a fine spatial resolution during the eighteenth and nineteenth centuries.

## State-of-the-art

Malaria was widespread across Europe, including the Nordic countries, until the mid to late nineteenth century, and was in Sweden especially prevalent in coastal areas [18, 48]. Although clustering of malaria was often seen among construction workers of railroads [23, 49], canals, dams and mining support activities, where they stayed in primitive workplaces often renowned for poor sanitary and overcrowded households [50], the presence of malaria vectors in the Scandinavian countries was identified to be climate-sensitive [51, 52]. In fact, the association between malaria epidemics and weather conditions was observed particularly with summer temperature and hydrological extremes [18, 43, 53]. Climate change-related components, especially the warming trend and certain extreme weather events such as heat-waves, are believed to increase the burden of vector-borne diseases [54, 55]. Temperature and precipitation are the two primarily, and most widely studied climate indicators in relation to malaria. Ambient temperature is crucial for sporogony development as well as a key driver in mosquito population dynamics. A temperature increase of a few degrees Celsius can result in an expanded geographical range of *P. vivax* beyond their usual limits in the north, and a larger population size of infectious *Anopheles* mosquitoes [44]. Precipitation variations, correspondingly, alter the aquatic habitats for mosquitoes and predators, but the evidence associated with precipitation is quite mixed and context-specific [56]. Clearly, the relationship between vectors and environmental factors is significant but often shaped in complicated mechanisms. Multiple climate variables as a better explanatory indicator is needed from a biological perspective [57, 58].

The decline of malaria in Europe has been entirely attributed to factors other than climate. At the same time, tracking the change in climate suitability in areas of potential risk for malaria transmission is of global importance [59]. The uncertainties regarding vector-borne diseases affected by climatic drivers highlight the need for local datasets covering long time spans, to develop better empirical models [60], and allow to quantify the assessment of the sensitivity of the disease in relation to both climatic and non-climatic factors [61]. Moreover, to capture heterogeneity at a regional scale which often poses a great location-specific sensitivity to climate has been a challenge in modeling climate–disease relationship [62, 63]. In light of a possible geographical shift in malaria incidence due to the ongoing anthropogenic climate change, gaining better knowledge of past climate–malaria relationship is of great relevance, and may even have policy implications for other climate-sensitive infectious disease surveillance at national level.

## Methods

### Data assembly

Obtaining historical primary source material that addresses diseases or mortality in a particular disease is challenging, especially records covering more distant time periods. To study the long-term variations of a disease, data from different sources are commonly combined to overcome the limits set by the short data coverage and/or an uneven geographical distribution of the source material. In this study, cases of malaria-attributed deaths in Sweden are mainly derived from data obtained from digitized parish records and from two older publications. Following an exploratory data analysis, the following three data sources were selected for this study:

1. The Tabellverket (1749–1859) dataset: Under the name Tabellverket, vital statistics, including the cause of death, were collected in Sweden from 1749 to 1859 and registered at parish level. As government officials, the priests of the Lutheran Church of Sweden were responsible for filling out the forms for their parish [64]. Several different forms were given out between 1749 and 1859, and the causes of death were specified in a list, containing between 33 and 41 categories, depending on the form used at the time. Regarding malaria, the Swedish term *frossa*, with various spellings, or the more symptomatic descriptive term *remittent fevers* was used to describe malaria. However, for the 1831–1859 period the causes of death had to be written down in the priests own wording for most diseases [65]. From 1860 onwards, routines changed and physicians were to write death certificates for all deaths in cities, while priests still reported the causes of death in the countryside. Reports on deaths from certain common diseases were then communicated to the newly formed government agency Statistics Sweden (*Statistiska centralbyrån*). Reported deaths by these diseases were then summarised at county level in official yearly publications, and one of the diseases listed in the publications was malaria [66]. Analysing causes of death in historic times in Sweden back to 1749 were facilitated by the digitalisation of all surviving Tabellverket forms by the Demographic Database (DDB) at Umeå University. However, comparisons between the DDB digitized data from Tabellverket and the statistical summaries from the time the original data was collected show that the digitized data from Tabellverket under-register death rates, most likely because all forms of vital statistics have not survived throughout the years [67]. It should be noted that several causes of death often were listed together on the pre-printed forms. This could be a problem in specific cases, but less so on an aggregated level. Besides, for the form used during 1821–1830, only *remittent fever* was assigned in the category as a collective name for diseases with symptoms of fever. During this ten-year period, there were 33,142 cases with 32,889 cases of *remittent fever* and 253 cases of *frossa* respectively, the latter was probably written down in a separate row available. Since sources from the military hospital in Stockholm (Flensburg’s dataset) showed more than 800 malaria cases recorded during the peak years 1828–1829, it is obvious that 253 *frossa* (malaria) cases in the entire country in ten years (1821–1830) cannot be correct. Furthermore, both datasets showed very similar peaks. For the above-mentioned reasons, “*remittent fever*” was included in this study to reflect the estimation of possible malaria cases 1821–1830, although they are likely to be overestimated as malaria-attributed deaths since they could not be easily distinguished from other diseases with symptoms of fever. In spite of the shortcomings, the digitized data from Tabellverket can still be considered a great asset for assessing the cause of death at parish level from 1749 to 1859 and to track the changes over time in the frequency of death in common diseases. In total, 90,178 cases of malaria deaths (i.e. symptoms typically described in Swedish as “*fråssa*”, “*frossa, omväxlande feber*”, “*frosse*” and “*remittente febrar*” were extracted from the Tabellverket database over the 1749–1859 period [68].
2. The Bergman (1749–1820) dataset: A table of mortality attributed to malaria in Sweden was published by the Swedish physician Gustaf Bergman [53]. This table combined data from Tabellverket, with some periods commonly shared by Sweden and present-day Finland prior to 1809 (when Finland still was still part of Sweden). The Bergman [53] data was digitized for the purpose of comparison with the Tabellverket data, digitized by the Demographic Database (DDB) at Umeå University, and to supplement it.
3. The Flensburg (1826–1890) dataset: A total number of 10,443 malaria cases were collected by Dr. Carl Flensburg from the military hospital in Stockholm [43]. These cases are malaria patients who had been treated in the hospital. It did thus not include patients not treated in the hospital. The data were digitized from Flensburg’s original article [43].

### The *frossa* diagnosis

As mentioned above, for a long time, *frossa* was the common name for malaria in Sweden. For example, the Swedish cause of death nomenclature from 1911 states that *Frossa (Febris intermittens)* should be written in official statistics, while the associated list for doctors stated the Latin equivalent *malaria* for the same disease [25]. The Swedish disease name *frossa* then existed alongside its Latin translation in official nomenclature up until the implementation of ICD–9 (International Classification of Diseases) in 1987 [69]. Before that, however, it had since long been replaced with malaria as an everyday expression for this disease. Prior to the discovery of the *Plasmodium* parasite in 1880, diagnosing was conducted through symptom observations. In general, *frossa* was divided into three different types, referring to how often the fever chills recurred, denoted as *quotidiana* (daily), *tertian* (every second day), *quartana* (every third day). The main symptoms associated with the diagnosis was chills and hot flushes [70]. However, the provincial physicians linked the *frossa* diagnosis with a wide spectrum of symptoms. Apart from irregular fever flushes, the most frequently observed symptoms were headaches, nausea, joint pains and diarrhea. Less frequently, it was associated with respiratory symptoms, and one physician associated dropsy and abdominal infarctions with malaria [71]. Moreover, the use of quinine to treat malaria seemed to have been fairly widespread in the mid-nineteenth century [72]. However, already in early-eighteenth century writings on malaria, the quinine treatment was mentioned in Swedish publications [70]. It was noticed that sickness in malaria was higher in areas with marshland and swamps. As modern medical theories did not emerge until the late nineteenth century, physicians adopted their observations into the theory of miasma, taking on that pathogenic vapours emerging from the wetlands caused the disease [73].

### Historical meteorological data

Monthly temperature data from Stockholm (1756–1890) [74] and Uppsala (1722–1890) [75] were used for comparison with the three malaria datasets. The Stockholm and Uppsala stations are approximately 70 km apart from each other, and they show similar summer temperature fluctuations (r_p_ = 0.93 during 1826–1890 for June–July). Temperature data from both places were homogenised, but an additional adjustment was applied to the Stockholm data to eliminate the warm bias of the thermometer’s exposure to solar radiation [76]. Monthly precipitation data from Stockholm (1756–1890) and Uppsala (1722–1890) were obtained from the Swedish Meteorological and Hydrological Institute (SMHI) [77].

The malaria time-series and the meteorological data were all standardized over their respective periods to show anomalies. The standardized value was calculated using the following formula:

$$\begin{aligned} Standardized\;value = \frac{X - \mu }{\sigma } \end{aligned}$$

where:

$$\begin{aligned} X =\hbox { the value of an observation}\\ \mu =\hbox { the mean}\\ \sigma =\hbox { the standard deviation} \end{aligned}$$

### Georeferencing the data

After the data of malaria-attributed deaths were collected from the digitized data from Tabellverket, they were sorted and summarised by each parish and year using Feature Manipulation Engine (FME) version 2020 by Safe Software [78]. The conversion of malaria records to spatially resolved data was achieved by matching the existing parish name with the historical parish georeferenced data from the Swedish National Archives (Riksarkivet). The closest matches were first automatically identified using the function *levenshtein* in the open source Geographic Information Systems (GIS) software QGIS [79], and a few identified mismatches were corrected through additional manual inspections by the authors.

### Mapping of annual malaria-attributed deaths

Annual malaria-attributed deaths from each parish in the digitized georeferenced data from Tabellverket were used to produce heat maps to demonstrate the spatial-temporal variations in the number of malaria-attributed deaths. This was performed using the built-in heat-map function in QGIS [79]. By defining a circular neighbourhood around the center of each data point, the programme will find the number of the points within this neighborhood, creating a density raster of these data points by interpolation (kernel density estimation) according to the colour ramp user customized. For the data used in this study, each data point was given the same weight regardless of the number of deaths reported from the same parish, which allows to emphasise the variations in spatial pattern instead of the strength of respective outbreaks. A radius of 25 kilometers was specified for visualizing a more generalized density pattern from year to year. The higher number of data points found in a particular area can be viewed as an area with higher malaria-attributed death density.

### Correlation analysis

Both Pearson (r_p_) and Spearman rank (r_s_) correlation analysis were performed to analyze the correlation between the number of malaria-attributed deaths and monthly and seasonal temperature and precipitation data. Pearson correlation is better suited to evaluate linear relationships between two variables, while Spearman rank correlation analysis is performed based on the ranked values for each variable, with a better ability to measure non-linear responses between variables. Spearman rank correlation was considered to be better suited especially in cases of extremely high numbers of malaria-attributed death during severe epidemics.

### Superposed Epoch Analysis

Superposed Epoch Analysis (SEA) [80–82] was employed to test whether anomalous climate conditions had a significant impact in years with particularly high numbers of malaria-attributed deaths. Standardized temperature anomalies, calculated by dividing the standard deviation (SD) for all event years, were compared to the five years prior to, and following, the years with most recorded malaria deaths/cases. The SEA was performed using a custom developed program written in F77-Fortran, and the 0.05 significance level was estimated through 1000 randomisations, and also made several tests of the malaria key year selection for the outcome. The Gaussian mean values from the SEA are presented, but it was found in an exploratory data analysis that the results are similar to using the median and the bi-weight robust means [83].

## Results

**Fig. 1:**
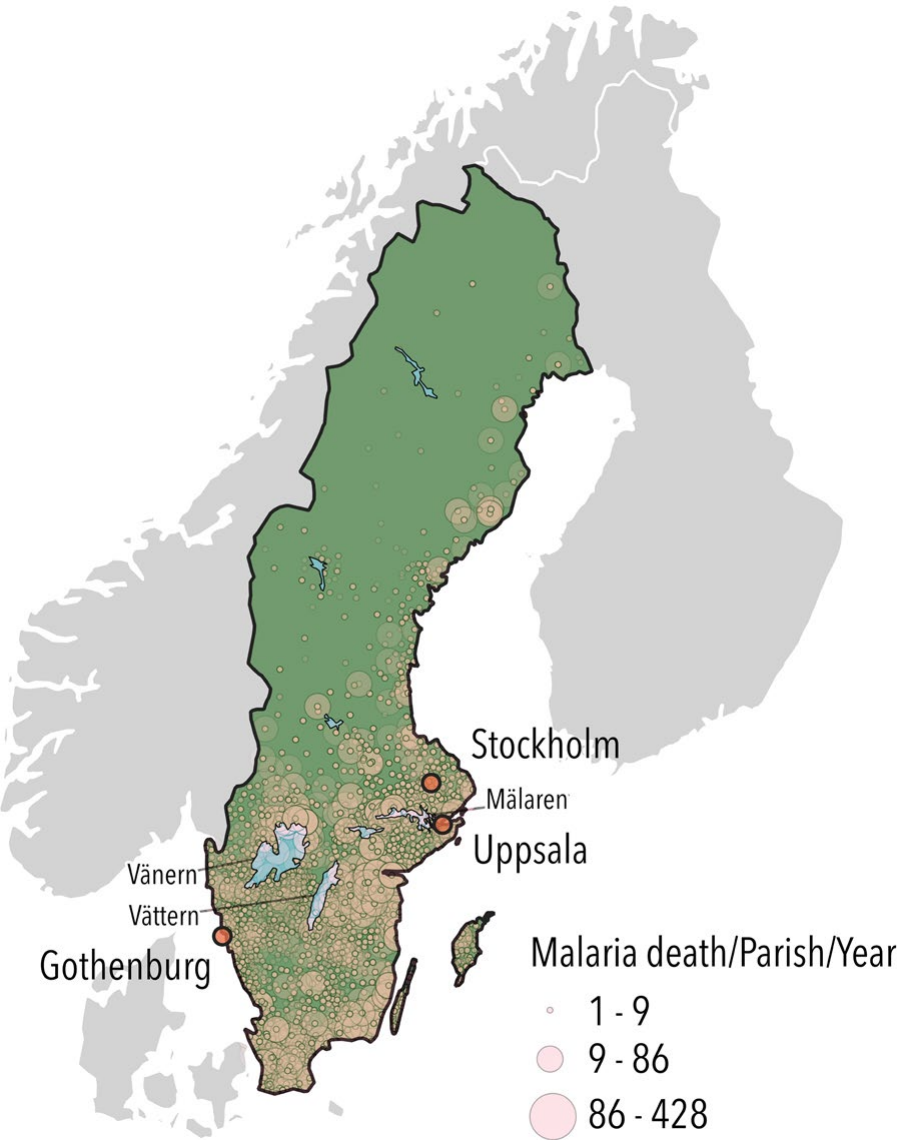
The spatial distribution of annual malaria-attributed deaths at parish level in Sweden. Each circle represents annual malaria-attributed deaths reported at a parish level 1749–1859. The size of the circle reflects the number of death cases

**Fig. 2:**
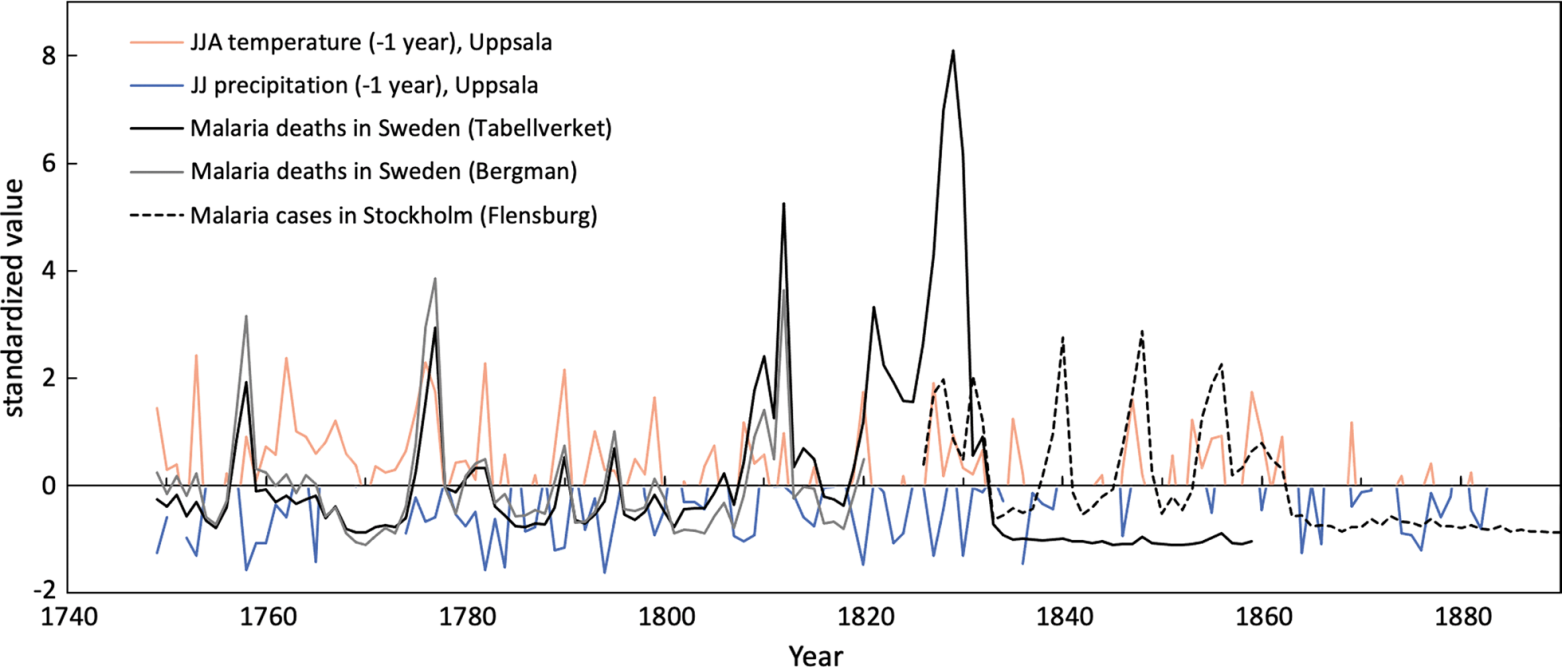
Time-series of malaria datasets against climate variables. Malaria datasets from three data sources (Tabellverket 1749–1859, Bergman 1749–1820, Flensburg Stockholm 1826–1890) and the relations to meteorological data. Malaria-attributed deaths of in the datasets from Tabellverket and Bergman represents the entire country, while Flensburg’s malaria cases were from the capital city, Stockholm. The meteorological data was moved backward with one year (–1). Only positive values of temperature data (orange line) and negative values of precipitation data (blue line) are shown to emphasise the correlations against the malaria datasets. Malaria-attributed deaths in the datasets of Tabellverket and Bergman have been standardized to their common period 1749–1820; the other series are standardized over their own respective period for the purpose of comparison. The drop in the number of malaria-attributed deaths after 1831 in the datasets from Tabellverket is a result of the way death registering had changed, the details are described in the Method section

The spatial distribution of the 90,178 cases of malaria-attributed deaths in Sweden between 1749 and 1859, retrieved from the digitized Tabellverket dataset compiled at parish level is presented in Fig. 1. The map shows that malaria-attributed deaths, and thus presumably transmission hot-spots, were located mainly on the eastern, but also western coastal areas, around large inland lakes, and in the southern-most part of Sweden. Combined with the data of Bergman and Flensburg, more than one hundred years of variations in malaria cases/deaths in Sweden during 1749–1859 are presented in Fig. 2. The most severe nationwide epidemic period occurred 1827–1830, with malaria-attributed deaths reaching almost 20,000 cases. Other notable time periods with elevated malaria cases/deaths include 1758, 1776–1777, 1810, 1812 and 1821–1822. Severe malaria outbreaks in the capital city of Stockholm can be seen in the Flensburg data in 1827–1828, 1831, 1840, 1847–1848 and 1854–1857.

### Climatic drivers

The climatic parameter and season associated with increased frequency of malaria were identified by means of Pearson and Spearman’s rank correlation analyses (Table 1). Positive correlations were found with the May–August temperature of the preceding year. The highest correlation was observed with July temperature of the preceding year, while the monthly combination with the strongest correlation between malaria and temperature was June–July. Correlation with August temperature was less strong, but still significant in Flensburg’s dataset. This relationship is much weaker in the data from Tabellverket due to a sharp decrease in available data after 1831. Nevertheless, the calculation using the common period with Bergman’s dataset, which is limited to 1820, gives comparable results. Among different malaria datasets, the strongest correlation was found between Bergman’s series of malaria-attributed deaths and the mean temperature of July–August during the preceding year (r_s_ = 0.57, *p* < 0.01) over the period of 1756–1820. Precipitation, in contrast to temperature, generally showed negative correlations with the number of malaria-attributed deaths. The most significant correlations were found for June–July precipitation during the preceding year (r_p_/r_s_  = − 0.26/− 0.45, *p* < 0.05/0.01). The correlations with May–July (MJJ) and June–August (JJA) precipitation were only found significant in some of the datasets using Spearman’s rank correlation, implying a more non-linear relationship.

**Table 1:** Pearson’s correlations/Spearman’s rank correlations (r_p_/r_s_) between the datasets from Tabellverket (1749–1859), Bergman (1749–1820), Flensburg (1826–1890) and monthly meteorological data of the preceding year from Stockholm (1756–1890) and Uppsala (1748–1890)

Dataset	Tabellverket		Bergman		Flensburg	
	Stockholm 1756–1859 (104 years)	Uppsala 1749–1859 (111 years)	Stockholm 1756–1820 (65 years)	Uppsala 1749–1820 (72 years)	Stockholm 1826–1890 (65 years)	Uppsala 1826–1890 (65 years)
Temperature						
Apr (– 1)	− 0.06/0.06	0.00/0.11	− 0.04/− 0.07	− 0.09/− 0.09	− 0.15/− 0.06	− 0.02/0.05
May (– 1)	0.15/0.12	0.16/0.16	0.20/0.25	0.04/0.14	0.14/0.13	0.18/0.18
** Jun** (– 1)	**0.27**/**0.20**	**0.24**/**0.21**	0.48*/0.47*	0.35*/0.35*	**0.30**/**0.30**	0.37*/0.40*
** Jul** (– 1)	0.15/**0.25**	0.16*0.31	0.46*/0.49*	0.42*/0.47*	0.37*/**0.30**	0.46*/0.40*
Aug (– 1)	− 0.02/0.09	0.01/0.09	0.20/**0.28**	0.24/0.23	**0.27**/**0.26**	0.32*/0.35*
** JJ** (– 1)	0.24/*0.25	0.23/0.31*	0.53*/0.52*	0.47*/0.47*	0.40*/0.35*	0.50*/0.50*
AMJ (– 1)	0.15/0.16	0.17/**0.22**	**0.26**/**0.26**	0.10/0.17	0.14/0.12	0.24/**0.25**
** MJJ** (– 1)	**0.24**/**0.23**	**0.25**/0.30*	0.48*/0.50*	0.34*/0.39*	0.37*/0.32*	0.46*/0.45*
** JJA** (– 1)	0.17/**0.21**	0.18/**0.25**	0.51*/0.57*	0.47*/0.47*	0.42*/0.40*	0.52*/0.54*
Precipitation						
Mar (– 1)	− 0.15/− 0.15	− 0.09/− 0.27*	− 0.23/−0.30	− 0.07/− 0.03	− 0.16/**− 0.26**	− 0.15/− 0.11
Apr (– 1)	0.10/0.13	0.09/−0.04	0.20/0.09	0.33*/0.23	− 0.11/− 0.11	− 0.02/0.11
May (– 1)	− 0.05/0.05	0.08/0.00	− 0.06/− 0.07	**0.29**/0.03	− 0.07/− 0.14	− 0.05/− 0.06
Jun (– 1)	− 0.16/− 0.11	**− 0.22**/− 0.35*	− 0.11/−0.20	**− 0.25**/− 0.44*	− 0.20/− 0.24	− 0.16/− 0.14
Jul (– 1)	− 0.07/0.00	− 0.11/− 0.30*	− 0.19/− 0.29	− 0.12/− 0.22	− 0.20/**− 0.27**	− 0.08− 0.03
Aug (– 1)	0.01/−0.04	− 0.03/− 0.17	0.14/0.15	0.00/−0.12	0.07/0.03	0.10/0.08
** JJ (– 1)**	− 0.15/− 0.03	**− 0.23**/− 0.44*	− 0.20/−0.26	**− 0.26**/− 0.45*	**− 0.28**/− 0.37*	− 0.15/− 0.14
MAM (– 1)	− 0.04/0.05	0.02/− 0.12	0.077−0.14	0.32*/0.16	− 0.19/− 0.24	− 0.13/− 0.05
AMJ (– 1)	− 0.09/0.01	− 0.05/**− 0.21**	0.05/− 0.03	0.14/− 0.12	**− 0.25**/**− 0.27**	− 0.14/− 0.04
MJJ (– 1)	− 0.13/0.01	− 0.15/− 0.34*	− 0.11/− 0.22	− 0.10/− 0.34*	**− 0.28**/− 0.39*	− 0.14/− 0.16
JJA (– 1)	− 0.10/− 0.01	− 0.17/− 0.44*	− 0.05/− 0.03	− 0.19/− 0.37*	− 0.16/**− 0.25**	− 0.04/− 0.05

Bold: *p* < 0.05 significant

*p < 0.01 highly significant

### Effect of anomalously warm temperatures

**Fig. 3:**
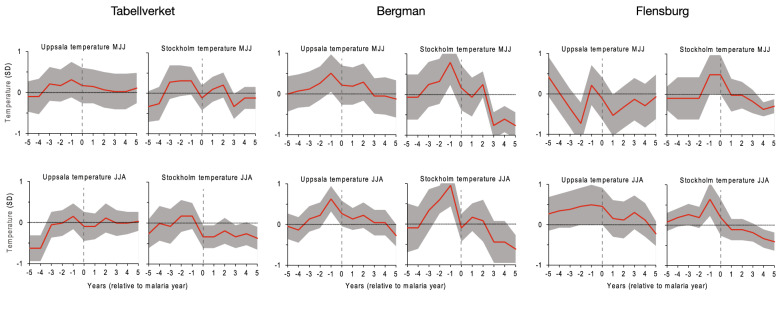
Superposed Epoch Analysis (SEA). Results from the Superposed Epoch Analysis (SEA) for three malaria datasets (Tabellverket, Bergman, and Flensburg), assessing the response of years with the 20 most malaria-attributed deaths (Tabellverket, Bergman) and malaria cases (Flensburg) to warm-season (May–July, June–August) temperature based on all years in the 1749–1859 period. The grey-shaded area represents 95% confidence intervals derived through 1000 randomisation. The results indicate that the peak years of malaria-attributed deaths, or cases, were significantly associated with the preceding warm-season temperature. The magnitude of the climate anomalies are shown as standard deviations (SD)

The occurrence of severe malaria events in response to climate anomalies over the entire 1749–1859 period was further tested by Superposed Epoch Analysis (SEA) assessing the years with most recorded malaria-attributed deaths. The results indicate that the peak years of malaria-attributed deaths were significantly (confidence interval = 95%) associated with positive anomalies in temperature of May–July and June–August of the preceding year (Fig. 3).

### Spatial variations of severe malaria epidemics

**Fig. 4:**
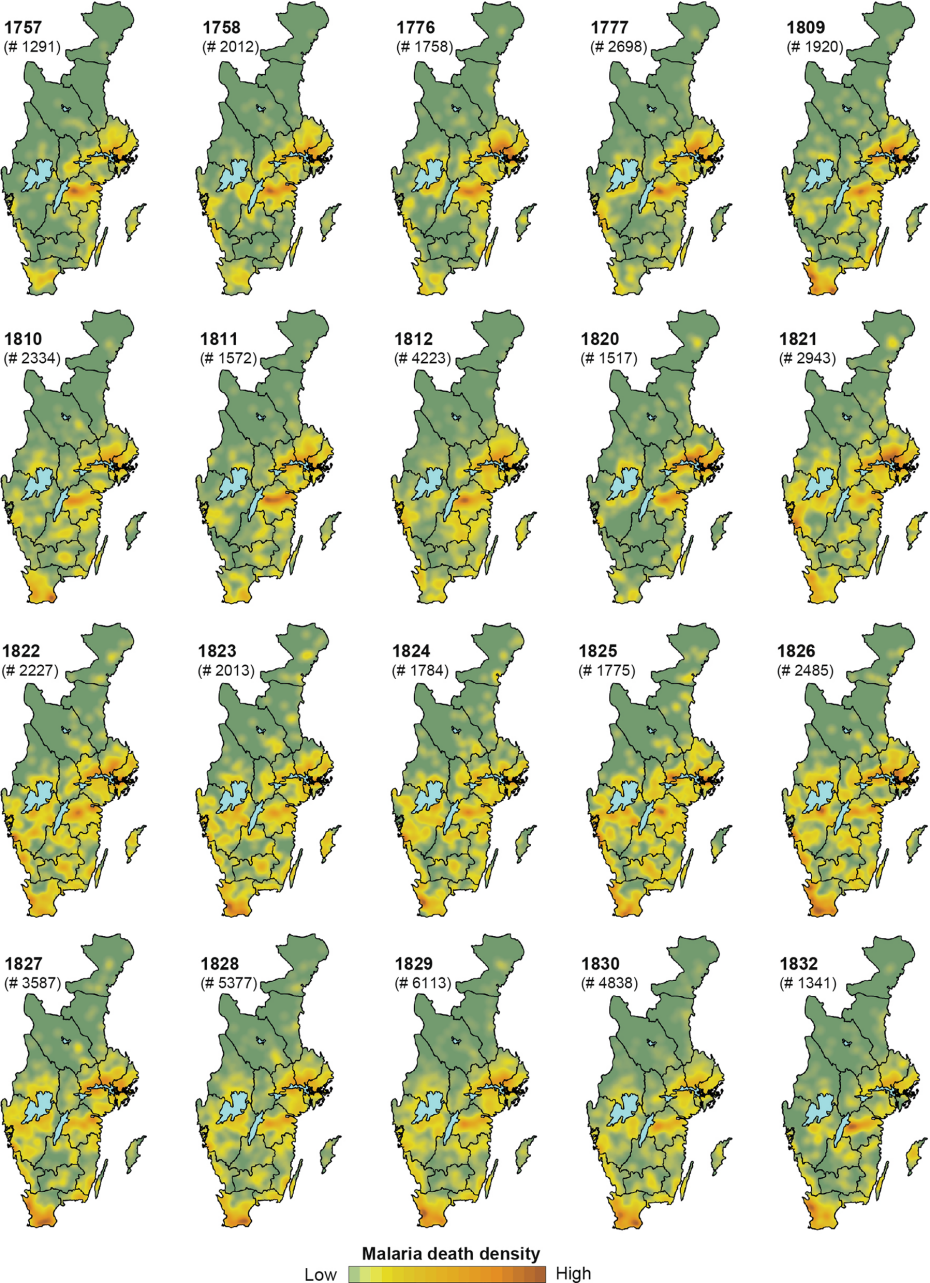
Spatial variations of the 20 years with most malaria-attributed deaths at parish level between 1749 and 1859. The heat-maps show the spatial-temporal distribution of malaria-attributed deaths at parish level during the 20 years with the most deaths attributed to malaria in Sweden using the digitized and georeferenced data from Tabellverket. The numbers of cases occur in a specific area are shown on a spectrum of green to red. Areas with warmer tones (yellow–orange) indicate higher malaria-attributed death density

The spatial distribution was evaluated, at parish level, of malaria-attributed deaths from Tabellverket during 1749–1859, the 20 years with the highest recorded deaths within the borders of present-day Sweden. The relative geographic distribution of malaria-attributed deaths is shown in heat maps with focus on the southern part of Sweden where most deaths were recorded. During these 20 years (Fig. 4), hot-spots were recognised around Lake Mälaren, Lake Vänern, and in big cities and their surroundings. To a certain extent the density of deaths is reflecting population density at the time. However, the region just north of Lake Mälaren and the plains of Östergötland, in the east, as well as Skåne in southern-most Sweden, show disproportionately high numbers of malaria-attributed deaths relative to these regions’ share of the population.

Each of those 20 years with the most recorded malaria-attributed deaths, representing the major malaria outbreak years in Sweden 1749–1859, reveal somewhat different geographical patterns of malaria mortality. The four major years with most malaria-attributed deaths in the eighteenth century—1757, 1758, 1776, and 1777—showed a concentration of malaria mortality around Lake Mälaren and in Östergötland, with only a modest mortality in likewise densely populated Skåne in the extreme south of the country. A similar pattern is evident for the early-nineteenth century years 1811, 1812, and 1820. Conversely the major centre of malaria-attributed deaths in 1830 and 1832 was in Skåne (Fig. 4).

During some years the malaria mortality was geographically confined to certain portions of Sweden, with little evidence of malaria mortality in other parts of the country. This was particularly the case 1757–1758, 1776–1777, and 1820. During other years, most notably 1822–1826, malaria-attributed deaths were geographically widespread across southern and central Sweden. In conclusion, the heat-map analysis reveals distinct patterns of malaria-attributed deaths, and thus presumably also the relative occurrence of malaria cases, shifting over time independent of the relative population density that remained comparable stable over the study period.

## Discussion

This study of malaria cases and deaths in eighteenth and nineteenth century Sweden allowed us to assess their associations with climate factors from a long-term perspective. The results from this study showed that the number of malaria deaths was positively associated with preceding summer temperatures (i.e. higher temperature = more deaths and vice versa), both when considering the inter-annual relationship and single extreme events. Malaria death distribution with a relatively high spatial resolution also demonstrated the transmission hot-spots varied geographically over time. The analysis also presented a number of challenges described as follows.

### Comparison of different datasets

In Bergman’s study, he used data over malaria-attributed deaths from Tabellverket, which should have yielded the same result as the digitized Tabellverket data employed in this study. However, before 1801 malaria-attributed deaths reported by Bergman’s death numbers were twice the number reported in the data from Tabellverket, while peak years with elevated malaria death were slightly lower in Bergman’s data. This discrepancy might be explained by the loss of records, and that this study have attributed slightly different causes as malaria deaths. Nevertheless, there is a very high correlation between the digitized data from Tabellverket and the data by Bergman (r_p_ = 0.88 for the common period 1749–1820), and the fluctuations are rather comparable between these two datasets. However, given the differences, it is considered appropriate to present both datasets on malaria deaths in this analysis.

### Missing data

In the official data from Tabellverket used in this analysis, for every year there is a lack of data from certain parishes. An estimated 20% of death records are missing in the digitized data by Tabellverket when compared to the contemporary official documentation at a national level [84]. For data of malaria-attributed deaths, a substantial part of the original data after 1830 is unfortunately missing. This can be attributed to the fact that only smallpox and neonatal death were specified on the form used during 1831–1859; other causes of deaths, including malaria, was not specified on the pre-printed forms. The priests therefore needed to report the cause of death manually. Hence, the data during the 1831–1859 period is incomplete. However, the decrease in malaria transmission by the end of the nineteenth century is evident from the data by Flensburg of malaria cases treated at the Stockholm military hospital. Since the number of military personnel was always 2700 men, his data is considered to show a reliable trend over time. Flensburg later complemented the data to include all malaria cases, including noted cases from the military barracks not treated in the hospital, but the continuity of this data varies between years. In this data (not shown), 2632 men were infected with malaria in 1828, which was consistent with the 1827–30 peak of malaria-related mortality in the data from Tabellverket. It is worth to again stress that the records on causes of death after 1831 were not complete, and Flensburg hence had to collect the data from *Sundhetskollegii årsberättelse* and from the publications of the Swedish Association of Medicine (*Svenska läkaresällskapets handlingar*) to compile the malaria cases in the entire country of Sweden between 1861 and 1909. His table shows 11,074 malaria cases were recorded in 1861, the most severe malaria year during this period. Other malaria years were 1862, 1873–1874, and 1877 during this period.

The temperature and precipitation data from Stockholm and Uppsala used in this study are among the longest meteorological observations in the world [85], but the precipitation data is neither continuous nor as reliable as the temperature data. In particular, the precipitation data from Stockholm 1859–1862, 1864–1870, 1880–1890, and from Uppsala 1863–1868 and 1878–1890 contain some questionable values. Monthly data from Uppsala is also occasionally missing in 1764–1773. Besides the concern about data continuity, the Stockholm temperature data displays considerably lower summer temperature values than Uppsala in the period prior to 1859, likely due to the warm bias adjustment in the process of homogenisation. This could be one explanation to why Flensburg’s malaria data from Stockholm shows higher correlation with temperature data from Uppsala compared to Stockholm (as would be expected).

### Malaria dynamics and summer temperatures

The time required for the maturation of the sporozoites in the mosquitoes depends on the accumulated time of temperature above the minimum threshold of 14.5 °C for *P. vivax* [86]. This limitation results in a very short window for parasite development in the mosquito and can vary locally. In southern Sweden, offspring from the hibernating *An. messeae* generation usually emerge as early as in May/June, depending on temperature and take blood meals during the summer months [34]. If the summer temperatures are high enough (e.g. in average 16 °C in June–August during study period 1749–1859), any malaria parasites taken up by these females during blood feeding may be able to complete their developmental cycle and mature parasites can be transmitted by the end of summer. In general, unusually warm summers are not necessarily connected to severe malaria epidemics, but the start of several major epidemic outbreaks coincided with anomalously warm summers during the preceding one to two years. These epidemic years were 1758, 1776–1777, 1808–1812, 1809–1812, 1820–1830, 1847–1848, and 1854–1856 (Fig. 2). This might suggest that the effect of increase in temperature cannot be translated into increased transmission potential, and this non-linearity of malaria dynamics in response to environmental factors like temperature is often found in malaria-endemic regions [87, 88].

### Malaria dynamics and hydrological variability

In addition to temperature being an important determinant of its dynamics, malaria is closely linked with the presence of water bodies. To a large extent, the high density of malaria-attributed deaths around large inland lakes and coastal regions of southern Sweden corresponds to the distribution of *Anopheles maculipennis* spp. vectors [18]. The association of malaria spreading with individual weather events is, however, elusive. A connection between malaria years, precipitation and the water level in Lake Mälaren was proposed by Bergman, suggesting that malaria epidemics usually were preceded by one or several years with unusually high rainfall and high lake levels, followed by warm summers often accompanied with droughts [53]. Since the Swedish *Anopheles* only lay eggs in permanent water, the hypothesis that they benefits from temporary water bodies during drought events cannot be supported. Although the correlation analyses failed to show a consistent link between malaria and precipitation, the results from the Spearman’s rank correlation show stronger correlations between precipitation and malaria compared to Pearson’s correlation analysis. This indicates that the influence of precipitation on malaria may not be linear. Furthermore, the dependent relationship of temperature and rainfall (Pearson correlation = − 0.4, 1748–1890, *n* = 143, from Uppsala meteorological station) makes it intractable to clarify the interplay of each factor associated with the number of malaria-attributed deaths. Interestingly, the most severe epidemic period 1820–1830, with a total of 34,659 malaria-attributed deaths in Sweden, coincided with four exceptionally dry years, but not always with warm summers. In short, considering all combined factors, simple correlative methods may not suffice when studying the response of malaria to some climate variables [89], especially if the response is non-linear. Approaches which are able to analyse the causation in a non-linear dynamic system are thus needed in future research to better evaluate the non-linear patterns in climate-associated malaria incidence.

### Comparison with previous studies

The early malaria studies in Sweden, conducted by Bergman [53] and Flensburg [43], provide extremely valuable information regarding the association between malaria epidemics and climate dynamics. Their pioneering attempts have helped to clarify the causation based on empirical observations. Bergman used maps to demonstrate the temporal delay of epidemic spread from coastal areas towards inland areas, and towards the north, during certain epidemic periods at a district level. This pattern of the temporal delay is, nevertheless, not distinct during earlier epidemics periods (1749–1831) in the spatial analysis (Fig. 4). Areas of intense transmission in Sweden and Finland were further mapped in Ekblom (1945) [18], in which malaria occurred mainly in the southeast of Sweden, but without such a fine spatial resolution as here. By analysing malaria-attributed deaths at a higher spatial resolution (i.e. parish level), it is made possible to detect that malaria was more prevalent in the southern-most province of Sweden (Skåne) in the nineteenth century compared to the late eighteenth century. During the major outbreak of 1827–1830 in southern Sweden, Denmark similarly saw outbreaks with over 4,235 malaria-attributed deaths [90] in eastern Denmark bordering southern Sweden (Skåne), known for having harboured malaria [91]. Furthermore, records of malaria from the military hospital in Stockholm (the Flensburg dataset) also present peak years estimates comparable with those in southern Finland [29], including the major peak years 1831, 1846–1847 (1847–1848 in Sweden) and 1854–1855 (1854–1857 in Sweden). This shows that severe malaria epidemics were widespread events. In addition, although different malaria datasets have been used, covering different time periods, the results from this study are consistent with earlier studies for Finland [29, 37]. This lends support to the conclusion that the summer temperature during the preceding year had a strong influence on malaria transmission. The use of the Spearman’s rank correlation in this study helped to reduce the effect of strong peaks in malaria mortality. This method is also suited to address non-linear relationships, allowing us to carry out an analysis of both the relationships between malaria and temperature and precipitation. The latter climate variable, showing a more non-linear effect, was not included in the studies for Finland [29, 37].

## Conclusions

Increased transmission of malaria in Sweden, and elsewhere in northern Europe, has historically been linked to higher average temperature during the summer of the preceding year. This study of malaria in Sweden during the eighteenth and nineteenth centuries supports such a relationship, i.e. that warm and/or dry summers may have contributed to more severe malaria outbreaks in the following year(s). Likely the warmer than usual temperatures increased the number of malaria vectors, and that symptoms of malaria infection appeared and were documented the following year, due to both indoor winter transmission and the evidenced long incubation and relapse time of the *P. vivax* malaria parasite. The effect of precipitation fluctuations on malaria was, on the other hand, found to be less significant, and likely being non-linear. Malaria hot-spots were concentrated in the areas around Lake Mälaren and the plains of Östergötland in the middle of the eighteenth century, but subsequently became more widespread across southern Sweden, especially in 1822–1826. Moreover, the outbreaks were generally more pronounced in Sweden’s southern-most region (Skåne) in the first half of the nineteenth century. These findings improve the knowledge of the history of malaria in Sweden, but also highlight the potential need for modelling malaria’s response to climate in a nonlinear dynamical system. The digitized and georeferenced data of malaria-attributed deaths in Sweden, and its distribution at a parish level, allows for further investigations of the dynamics behind malaria outbreaks and the shift of transmission hot-spots with emphasis on possible controlling demographic, social, and economic factors.

## Data Availability

The data that support the findings of this study are available from Centre for Demographic and Ageing Research (CEDAR), Umeå University, but restrictions apply to the availability of these data, which were used under license for the current study, and so are not publicly available. Data are however available from the authors upon reasonable request and with permission of the Centre for Demographic and Ageing Research (CEDAR).

## Abbreviations

GIS: Geographic Information Systems
FME: Feature Manipulation Engine
SEA: Superposed Epoch Analysis
SD: standard deviation
